# Generalized problematic internet use: An impulsive-compulsive spectrum disorder?

**DOI:** 10.1192/j.eurpsy.2021.1519

**Published:** 2021-08-13

**Authors:** A. Araujo, A.T. Pereira, M.J. Soares, B. Rodrigues Maia, A. Macedo

**Affiliations:** 1 Institute Of Psychological Medicine, Faculty of Medicine, University of Coimbra, Coimbra, Portugal; 2 Faculty Of Philosophy And Social Sciences, Centre For Philosophical And Humanistic Studies, Portugal, Universidade Católica Portuguesa, Braga, Portugal

## Abstract

**Introduction:**

Generalized problematic internet use/GPIU has recently been associated with the impulsive-compulsive spectrum/ICS, but its mapping onto these behaviour dimensions is relatively unexplored.

**Objectives:**

To compare patterns of internet use and scores of BIG-5 personality traits, perfectionism and psychological distress between groups with low/high levels of GPIU.

**Methods:**

475 university students (78.9% girls; mean age 20.22±1.695) answered the Portuguese versions of: GPIU Scale, Multidimensional Perfectionism Scale-13, NEO-FFI-20, Depression, Anxiety and Stress Scales and other questions about internet use. Chi-square and Mann-Whitney tests were performed using SPSS.

**Results:**

Individuals with high levels of GPIU (median+2SD; n=18; 3.8%) spent significantly more time/day in online activities, exceeding what they have planned; had no other hobbies and used social networks to meet friends; reported that GPIU interfered with affective/work relationships and academic performance (all p<.05). There were no significant differences in the purposes of the internet use (e-mail, social networks, shopping, videogames, multimedia, sexual, work…), unless for general information searching and betting games (both p<.05). High-PGIU group also presented significant higher levels of neuroticism, negative (but not positive) perfectionism, depression, anxiety, and stress (all p<.001).

**Conclusions:**

Our results indicate that unlike the purposes of internet use, personality, perceived interference and the associated cognitive-emotional processes and symptoms (psychological distress) may help distinguishing between functional vs. dysfunctional internet use. Considering the preponderance of processes over contents and the presence of certain dimensions, such as perception of uncontrollability, interference and social isolation we add more evidence to consider PGIU as falling within the spectrum of impulsive-compulsive disorders.

**Conflict of interest:**

No significant relationships.

